# Chemotherapy-induced febrile neutropenia (FN): healthcare resource utilization (HCRU) and costs in commercially insured patients in the US

**DOI:** 10.1007/s00520-024-08492-5

**Published:** 2024-05-23

**Authors:** Jeanine A. Flanigan, Marie Yasuda, Chi-Chang Chen, Edward C. Li

**Affiliations:** 1 University of Pittsburgh School of Pharmacy, Pittsburgh, PA USA; 2 IQVIA Inc, Plymouth Meeting, PA USA; 3 Sandoz Inc, Princeton, NJ USA

**Keywords:** Febrile neutropenia, chemotherapy, healthcare costs, neutropenia, febrile neutropenia episodes

## Abstract

**Purpose:**

Febrile neutropenia (FN) is a known side effect of chemotherapy, often requiring hospitalization. Economic burden increases with an FN episode and estimates of cost per episode should be updated from real-world data.

**Methods:**

A retrospective claims analysis of FN episodes in patients with non-myeloid malignancies from 2014 to 2021 was performed in IQVIA PharMetrics® Plus database. FN episodes were defined as having same-day claims for neutropenia and fever or infection, plus antibiotic in outpatient settings, following a claim for chemotherapy; index date was defined as the first claim for neutropenia/fever/infection. Patients receiving bone marrow/stem cell transplant and CAR-T therapy were excluded, as were select hematologic malignancies or COVID-19. Healthcare utilization and costs were evaluated and described overall, by episode type (w/wo hospitalization), index year, malignancy type, NCI comorbidity score, and age group.

**Results:**

7,033 FN episodes were identified from 6,825 patients. Most episodes had a hospitalization (91.2%) and 86% of patients had ≥1 risk factor for FN. Overall, FN episodes had a mean (SD) FN-related cost of $25,176 ($39,943). Episodes with hospitalization had higher average FN-related costs versus those without hospitalization ($26,868 vs $7,738), and costs increased with comorbidity score (NCI=0: $23,095; NCI >0-2: $26,084; NCI ≥2: $26,851).

**Conclusions:**

FN continues to be associated with significant economic burden, and varied by cancer type, comorbidity burden, and age. In this analysis, most FN episodes were not preceded by GCSF prophylaxis. The results of this study highlight the opportunity to utilize GCSF in appropriate oncology scenarios.

**Supplementary Information:**

The online version contains supplementary material available at 10.1007/s00520-024-08492-5.

## Introduction

Febrile neutropenia (FN) is a well-established, serious side effect of myelosuppressive chemotherapies, typically considered to be a medical emergency when occurring following chemotherapy administration [1, 2]. As FN episodes have a highly increased risk of infection, hospitalization is often required [1, 3]. An FN episode causes significant sequelae to patients, which may interrupt chemotherapy, and also represents a significant burden on the healthcare system with prolonged hospitalization stays involving wide ranges of resource costs per episode [3, 4].

The development of granulocyte colony-stimulating factors (GCSF) revolutionized the use of chemotherapy, reducing the incidence of severe neutropenia in patients receiving myelosuppressive chemotherapy, subsequently allowing more patients to receive adequate dose intensity chemotherapy, and led to improved overall survival. Accordingly, the National Comprehensive Cancer Network (NCCN) recommends all oncology patients be evaluated for their risk of developing FN prior to chemotherapy initiation, and categorize patients as low-risk (<10%), intermediate-risk (10%-20%), or high-risk (≥20%) for FN [2]. Guidelines state that GCSFs should be provided as primary prophylaxis in patients deemed high FN-risk, while this strategy should be considered in patients at intermediate and low FN-risk, based on presence of ≥1 and ≥2 risk factors (RF), respectively [2, 5].

However, guidelines historically only recommended GCSF prophylaxis if regimens had >40% FN risk; this was lowered to 20%, encompassing the entire high FN-risk category, based in-part due to economic modeling showing cost-minimization of total healthcare costs at this threshold [6, 7]. Furthermore, changes within the healthcare environment may warrant a reconsideration of these thresholds. First, biosimilar GCSFs were introduced in the United States (US) in 2017, offering the same high-quality care at a lower price point and improving access to care for cancer patients [8]. This decrease in cost of GCSF products have improved cost-effectiveness of primary prophylaxis strategies, with one study finding biosimilar (filgrastim-sndz) primary prophylaxis was cost-effective in patients with intermediate-risk for FN receiving certain chemotherapy regimens, suggesting future expansion of GCSF use in appropriate patients [9]. However, this study utilized older input estimates for the costs of FN episodes.

The current study was performed to assess real-world healthcare resource and costs associated with FN episodes in the US among patients receiving chemotherapy. This study aimed to describe the costs and trends in FN-related hospitalizations, emergency room visits, and GCSF use in all FN episodes, as well as RFs for the patient. Subgroups of malignancy type, age groups, and comorbidity groups were scrutinized separately for impact on the healthcare burden.

## Methods

### Study design and data source

This descriptive retrospective analysis of FN episodes utilized the IQVIA PharMetrics® Plus claims database from July 1, 2014, through December 31, 2021. The analysis was performed at the episode level, allowing multiple episodes per patient which produced more comprehensive estimation of costs and resources used.

IQVIA PharMetrics® Plus is a health plan claims database comprised of fully-adjudicated medical and pharmacy claims. The database is representative of the commercially insured US national population for patients under 65 years of age.

### Episode definition and identification

An FN episode was identified by either of the following definitions: 1) ≥1 inpatient medical claim with a diagnosis of neutropenia and with a diagnosis of fever, bacterial infection, or fungal infection on the same date, or 2) ≥1 outpatient medical claim with a diagnosis of neutropenia, and with a diagnosis of fever, bacterial infection, or fungal infection on the same date, as well as evidence of administration of an NCCN-recommended antibiotic on the same date [1]. NCCN-recommended antibiotics were defined as a pharmacy claim for ciprofloxacin with amoxicillin/clavulanate or clindamycin; levofloxacin or moxifloxacin; or administration of a parenteral antibiotic via HCPCS [10]. Upon identification, an episode was additionally required to have ≥1 pharmacy or medical procedure claim for chemotherapy or biologic medication within 30 days prior to the episode start date (see Supplemental Table 1a for medication list).

The index date for each episode was defined as the date of the first claim for neutropenia, infection, or fever during the episode. Episodes ended on either the date of next incident chemotherapy treatment or after 60 days, whichever occurred first. However, if the patient had a FN-related hospitalization during the episode period and the discharge date extended beyond 60 days past the index date, the entire inpatient length of stay (LOS) was included in analysis (Supplemental Table 1a and 1b). Each episode was required to come from patients with continuous enrollment during the 180-day period prior to the episode index date, considered the pre-episode period, and was used to assess clinically relevant comorbidities. The 30 days prior to the index date was considered the chemotherapy treatment period and was used to assess treatments utilized prior to FN episode. Episodes with ≥1 diagnosis code of neutropenia, infection, or fever prior to episode index date, ≥1 medical claim with a procedure code for bone marrow, stem cell transplant, or CAR-T cell therapy or ≥1 diagnosis code for acute lymphocytic leukemia, acute myeloid leukemia, chronic myelogenous leukemia, or myelodysplastic syndromes during the pre-episode period were excluded, as these treatments and diagnoses may imply hematological processes which can complicate interpretation of FN. A subsequent FN episode qualified only if there was an ≥30-day period between episodes without diagnoses of neutropenia, infection, or fever.

### Study outcomes

#### Demographic and clinical characteristics

Baseline patient demographics were collected on the episode index date, including age, sex, and payer type, as well as episode length in days. Baseline clinical characteristics were captured during the pre-episode period, including hematologic malignancies and solid tumor malignancies, cancer type by site, NCCN-defined FN-related risk factors (age ≥65 years, bone metastasis, surgery, radiation, severe liver dysfunction, and kidney dysfunction) [2], categories of National Cancer Institute (NCI) Charlson Comorbidity Index (CCI) score (10), and select comorbid conditions. Diagnoses and procedures were captured using ICD-10-CM diagnosis and procedure codes (Supplemental Table 2).

#### Treatments

**Fig. 1:**
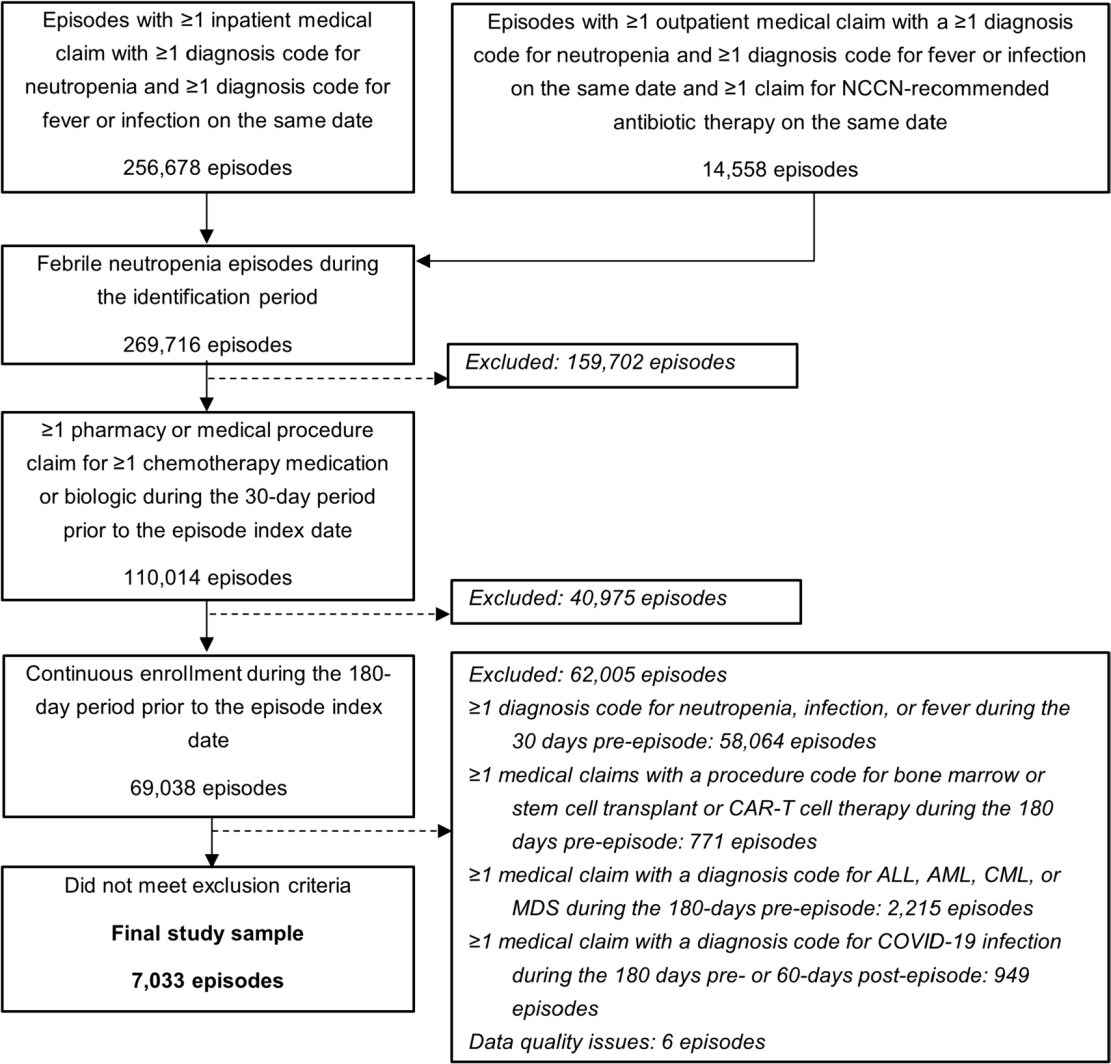
Attrition of the study sample. Abbreviations: *ALL* acute lymphoblastic leukemia, *AML* acute myeloid leukemia, *CAR-T* chimeric antigen receptor-T cell therapy, *COVID-19* coronavirus disease of 2019, *CML* chronic myeloid leukemia, *MDS* myelodysplastic syndromes, *NCCN* national comprehensive cancer network

Treatments were assessed during the chemotherapy treatment period; the chemotherapy regimen, GCSF treatments, and antimicrobial (antibacterial, antifungal, or antiviral) utilization were captured for each episode. Chemotherapy regimens were categorized into intermediate- and high-risk of developing FN, according to NCCN guidelines [2]; the remaining regimens were categorized as low/undefined. GCSF utilization and antimicrobial medications were separated into mutually-exclusive categories of prophylactic use or use in treatment, with prophylactic use defined as utilization within 4 days of chemotherapy administration [11]; all other utilization was considered used as treatment (see Fig. 1). GCSF utilizations were further categorized as short-acting GCSF or long-acting GCSF.

#### Healthcare resource variables

Healthcare resource utilization (HCRU) was evaluated for each episode, inclusive of the episode index date. Total all-cause utilization was split into inpatient hospitalizations, emergency room (ER) visits, physician office visits, other outpatient claims, and pharmacy claims. Costs were captured for each episode, presented as total costs and split into utilization categories.

FN-related utilization and costs were evaluated similarly during the episode period, defined as a medical claim with a diagnosis code for neutropenia, infection, or fever during the episode; FN treatments were defined as antimicrobials and GCSF administered/prescribed during the episode.

### Data analysis

Categorical demographic and clinical variables in the pre-episode and chemotherapy treatment periods were presented as the proportion of episodes with evidence of the specific variable (n, %); continuous variables are presented as the mean, standard deviation (SD), median, as well as minimum and maximum values for episode length. HCRU are presented as the number of episodes with evidence of utilization for each HCRU category, as well as mean, median, and SD number of HCRU for each category per episode. Costs among patients with a claim in that category during the episode were calculated in 2021 US dollars ($USD) and are presented as mean, median, and SD.

Study outcomes were assessed for all FN episodes, and by the following sub-cohorts: 1) Episodes that included ≥1 inpatient claim at any time during the episode period (‘Episode with hospitalization’); all others were considered an 'Episode without hospitalization’; 2) Episodes were stratified into 8 categories based on calendar year of the index date from 2014 to 2021; 3) Hematologic malignancies (Non-Hodgkin lymphoma [NHL], chronic lymphocytic leukemia [CLL], and other hematologic [e.g., Hodgkin lymphoma; lymphosarcoma; histiocytosis; mastocytosis; reticulosarcoma]); assessed as a composite group and as individual malignancies; 4) Solid tumor malignancies (breast, lung, colorectal, prostate, and other solid tumor; assessed as a composite group and as individual malignancies; 5) NCI CCI on index date (0, >0–2, or ≥2); and 6) age categories (<18, 18-59, 60-74, and ≥75 years).

## Results

### FN episodes

A total of 269,716 FN episodes were identified, and after applying all study criteria, final study sample consisted of 7,033 FN episodes (Fig. 1); 97.1% (*N* = 6,629) of patients had a single qualifying episode.

The majority of episodes had a hospitalization during the episode period (91.2%). Episodes were fairly evenly distributed across chronic comorbid disease severity levels, with 39.9%, 23.3%, and 36.8% having a comorbid burden score of 0, an NCI CCI score >0-2, and a score ≥2, respectively. For the number of episodes in all sub-cohorts, see Table 1.

**Table 1:** FN episodes by sub-group

Episode Subgroups	Episodes (*N*)
All patients	7,033
Episode Type*:	
Episodes with hospitalization	6,411 (91.2%)
Episodes without hospitalization	622 (8.8%)
Index Year*	
2014	6 (0.1%)
2015	473 (6.7%)
2016	1,485 (21.1%)
2017	1,365 (19.4%)
2018	1,331 (18.9%)
2019	1,285 (18.3%)
2020	906 (12.9%)
2021	182 (2.6%)
Hematological Malignancy	
NHL	1,010 (14.4%)
CLL	148 (2.1%)
Other hematologic	504 (7.2%)
Solid Tumor Malignancies	
Breast	1,919 (27.3%)
Lung	1,066 (15.2%)
Colorectal	781 (11.1%)
Prostate	252 (3.6%)
Other solid tumor	3,344 (47.6%)
NCI CCI Score*	
0	2,803 (39.9%)
>0 - >2	1,639 (23.3%)
≥2	2,591 (36.8%)
Age Category*	
<18	241 (3.4%)
18 to 59	3,580 (50.9%)
60 to 74	2,778 (39.5%)
≥75	434 (6.2%)

*Denotes groups within categories are mutually-exclusive

*CLL* Chronic lymphocytic leukemia, *NHL* Non-Hodgkin lymphoma

Across all episodes, the mean episode length was 32 days, with a median of 21 days. Episodes with hospitalization were longer (mean: 33 days, median: 22 days) than episodes without hospitalization (mean: 24 days, median: 15 days). However to note, episodes without hospitalization were capped at 60 days, whereas episodes with hospitalizations could extend longer than 60 days, with the maximum duration observed of 115 days. Episode length appeared to be stable across index years at approximately 32 days; however, episode lengths varied by cancer type; episodes with an NCI CCI score of ≥2 were longer (mean: 35 days, median: 26 days) than episodes with a chronic comorbidity score of 0 (mean: 29 days, median: 18 days).

### Pre-episode characteristics of Fn episodes

#### Demographic and Clinical Characteristics

Across all episodes, the mean age was 55.4 years; just 3.4% occurred in <18 years age group. The majority of episodes came from female patients (60.6%); distribution by sex was similar across episode hospitalization type, most index years and comorbidity score categories. Commercial was the most common payer type (87.5%), with 7.4% Medicare coverage. See Table 2 for full demographic and clinical information across all episodes (demographic information for sub-groups is available in Supplemental Table 3).

**Table 2:** Demographic and clinical characteristics of overall FN episodes and hospitalization subgroups

Episode Characteristics	Episodes (*N*=7,033)	Episode type: With hospitalization (*N*=6,411)	Episode type: Without hospitalization (*N*=622)
Age, years	55.4 (15.0)	55.6 (14.9)	53.3 (15.6)
Mean (SD)	58	59	56
Median	55.4 (15.0)	55.6 (14.9)	53.3 (15.6)
Sex, n (%)			
Male	2,773 (39.4%)	2,540 (39.6%)	233 (37.5%)
Female	4,260 (60.6%)	3,871 (60.4%)	389 (62.5%)
Payer type, n (%)			
Commercial	6,157 (87.5%)	5,608 (87.5%)	549 (88.3%)
Medicare	518 (7.4%)	471 (7.3%)	47 (7.6%)
Medicaid	150 (2.1%)	139 (2.2%)	11 (1.8%)
Unknown	208 (3.0%)	193 (3.0%)	15 (2.4%)
Geographic region, n (%)			
Northeast	1,248 (17.7%)	1,168 (18.2%)	80 (12.9%)
Midwest	1,884 (26.8%)	1,713 (26.7%)	171 (27.5%)
South	2,815 (40.0%)	2,538 (39.6%)	277 (44.5%)
West	1,064 (15.1%)	971 (15.1%)	93 (15.0%)
Unknown	22 (0.3%)	21 (0.3%)	1 (0.2%)
Episode length, days			
Mean (SD)	31.8 (22)	32.5 (22)	24.1 (21)
Median (Min, Max)	21 (1, 115)	22 (1, 115)	15 (1, 60)
NCI score			
Mean (SD)	1.8 (1.9)	1.8 (1.9)	1.6 (1.8)
Median	1	2	1

For demographics and clinical characteristics of subgroups of hematologic malignancy, solid tumor types, NCI CCI score, and age category see Supplemental Table 2.

#### FN Risk Factors

Of all episodes, ≥86.0% had an observed RF for FN; all RF were assessed independently. Surgery was the most common RF, followed by radiation therapy (24.0%), age ≥65 years (21.3%), bone metastasis (17.0%), kidney dysfunction (14.2%), and severe liver dysfunction (3.3%). Among subgroups, radiation therapy was observed most frequently in lung cancer, with half of the 1,066 episodes (50.5%) receiving radiation in the pre-index period. Bone metastases were observed most frequently in prostate cancer, with over half of the 252 episodes (57.1%) having evidence of bone metastasis. See Table 3 for RFs across all episodes.

**Table 3:** Risk factors for febrile neutropenia (FN), overall and by sub-groups

NCCN-Defined Risk Factors for FN	Older age*	Bone metastasis	Surgery	Radiation	Severe liver dysfunction	Kidney dysfunction
All Episodes (*N*=7,033)	1,499 (21.3%)	1,192 (17.0%)	6,049 (86.0%)	1,691 (24.0%)	234 (3.3%)	996 (14.2%)
Episode Type						
With hospitalization (*N*=6,411)	1,381 (21.5%)	1,111 (17.3%)	5,510 (86.0%)	1,576 (24.6%)	225 (3.5%)	916 (14.3%)
Without hospitalization (*N*=622)	118 (19.0%)	81 (13.0%)	539 (86.7%)	115 (18.5%)	9 (1.5%)	80 (12.9%)
NCI CCI Score						
0 (*N*=2,803)	386 (13.8%)	434 (15.5%)	2350 (83.8%)	584 (20.8%)	14 (0.5%)	96 (3.4%)
>0 - >2 (*N*=1,639)	357 (21.8%)	253 (15.4%)	1405 (85.7%)	413 (25.2%)	18 (1.1%)	214 (13.1%)
≥2 (*N*=2,591)	756 (29.2%)	505 (19.5%)	2294 (88.5%)	694 (26.8%)	202 (7.8%)	686 (26.5%)
Hematological Malignancy						
NHL (*N*=1,010)	255 (25.2%)	103 (10.2%)	945 (93.6%)	79 (7.8%)	51 (5.0%)	177 (17.5%)
CLL (*N*=148)	45 (30.4%)	11 (7.4%)	122 (82.4%)	8 (5.4%)	11 (7.4%)	22 (14.9%)
Other (*N*=504)	113 (22.4%)	82 (16.3%)	421 (83.5%)	44 (8.7%)	17 (3.4%)	106 (21.0%)
Solid Tumor						
Breast (*N*=1,919)	283 (14.7%)	355 (18.5%)	1,770 (92.2%)	251 (13.1%)	33 (1.7%)	130 (6.8%)
Lung (*N*=1,066)	344 (32.3%)	332 (31.1%)	905 (84.9%)	538 (50.5%)	42 (3.9%)	187 (17.5%)
Colorectal (*N*=781)	163 (20.9%)	89 (11.4%)	652 (83.5%)	253 (32.4%)	28 (3.6%)	104 (13.3%)
Prostate (*N*=252)	121 (48.0%)	144 (57.1%)	185 (73.4%)	65 (25.8%)	8 (3.2%)	58 (23.0%)
Other (*N*=3,344)	712 (21.3%)	578 (17.3%)	2,891 (86.5%)	1,095 (32.7%)	133 (4.0%)	551 (16.5%)

*CLL* Chronic lymphocytic leukemia, *NHL* Non-Hodgkin lymphoma

* Older age is defined as age ≥65 years on the index date

### Treatments

#### Chemotherapy: FN risk classification determinant

Among all FN episodes, nearly half received a pre-episode chemotherapy regimen with intermediate FN-risk (49.4%) prior to episode index, while 19.7% received a high-risk chemotherapy regimen prior and the remaining 30.9% received a regimen with low/undefined FN-risk.

Episodes with breast cancer were more frequently preceded by chemotherapy regimens with known high FN-risk (51.2%; 33.9% intermediate risk; 14.9% low/undefined risk) as compared to other types of solid tumors. Episodes with NHL were less frequently preceded by chemotherapy regimens with known high FN-risk (14.1%).

#### Pre-episode GCSF use

2,812 episodes (40.0% of total episodes) had GCSF use during the chemotherapy treatment period, either as prophylaxis or treatment; of these, 82.8% utilized long-acting GCSF and 20.7% utilized short-acting GCSF. From 2015 to 2021, the percentage of FN episodes with GCSF prophylactic use modestly increased (23.7% and 28.6%). 67.2% of episodes with GCSF use qualified for prophylactic use and 58.4% of episodes with GCSF use were considered use treatment (these categories were not mutually exclusive; episodes could utilize GCSF multiple times). In episodes with prophylactic use, the vast majority (92.8%) utilized long-acting GCSF; in episodes with treatment use, approximately three-quarters (76.9%) of episodes utilized long-acting GCSF. Over half of episodes with breast cancer utilized GCSF prior to episode (55.2%); this utilization rate was substantially higher than lung (26.6%), colorectal (25.0%), and prostate cancers (24.6%) as well as other solid tumors (35.3%).

#### pre-episode period antimicrobial use

1,696 episodes (24.1% of total episodes) had antimicrobial use pre-episode; among them, 28.4% qualified as prophylactic use, and 79.3% were considered treatment use (not mutually exclusive, both types of antibiotics could be utilized).

For full pre-episode period chemotherapy, GCSF, and antimicrobial treatments across subgroups, see Table 4.

**Table 4:** Treatments during the chemotherapy treatment period of FN episodes, overall and by sub-groups

Episode Treatments^1^	Chemotherapy regimen classification for FN risk^2^			Episodes with CSF use						Episodes with antimicrobial use	
	High-risk	Intermediate-risk	Undefined risk	Any Use		Use as Prophylactic^3,4^		Use as treatment^3,4^		Use as Prophylactic^3,4^	Use as treatment^3,4^
				Long-acting GCSF	Short-acting GCSF	Long-acting GCSF	Short-acting GCSF	Long-acting GCSF	Short-acting GCSF		
All Episodes (N=7,033)	1,384 (19.7%)	3,475 (49.4%)	2,174 (30.9%)	2,327 (33.1%)	581 (8.3%)	1,754 (24.9%)	140 (2.0%)	1,263 (18.0%)	423 (6.0%)	482 (6.9%)	1,345 (19.1%)
Episode Type											
With hospitalization (N=6,411)	1,237 (19.3%)	3,170 (49.5%)	2,004 (31.3%)	2,133 (33.3%)	533 (8.3%)	1,601 (25.0%)	129 (2.0%)	1,163 (18.1%)	388 (6.1%)	439 (6.8%)	1,246 (19.4%)
Without hospitalization (N=622)	147 (23.6%)	305 (49.0%)	170 (27.3%)	194 (31.2%)	48 (7.7%)	153 (24.6%)	11 (1.8%)	100 (16.1%)	35 (5.6%)	43 (6.9%)	99 (15.9%)
NCI CCI Score											
0 (N=2,803)	722 (25.8%)	1,260 (45.0%)	821 (29.3%)	985 (35.1%)	244 (8.7%)	736 (26.3%)	62 (2.2%)	562 (20.0%)	178 (6.4%)	196 (7.0%)	534 (19.1%)
>0 - >2 (N=1,639)	317 (19.3%)	790 (48.2%)	532 (32.5%)	549 (33.5%)	140 (8.5%)	416 (25.4%)	27 (1.6%)	290 (17.7%)	97 (5.9%)	100 (6.1%)	339 (20.7%)
≥2 (N=2,591)	345 (13.3%)	1,425 (55.0%)	821 (31.7%)	793 (30.6%)	197 (7.6%)	602 (23.2%)	51 (2.0%)	411 (15.9%)	148 (5.7%)	186 (7.2%)	472 (18.2%)
Hematological Malignancy											
NHL (N=1,010)	142 (14.1%)	564 (55.8%)	304 (30.1%)	556 (55.0%)	108 (10.7%)	408 (40.4%)	25 (2.5%)	309 (30.6%)	79 (7.8%)	110 (10.9%)	253 (25.0%)
CLL (N=148)	11 (7.4%)	77 (52.0%)	60 (40.5%)	59 (39.9%)	17 (11.5%)	44 (29.7%)	4 (2.7%)	30 (20.3%)	11 (7.4%)	21 (14.2%)	38 (25.7%)
Other (N=504)	63 (12.5%)	165 (32.7%)	276 (54.8%)	122 (24.2%)	58 (11.5%)	85 (16.9%)	17 (3.4%)	65 (12.9%)	44 (8.7%)	65 (12.9%)	155 (30.8%)
Solid Tumor											
Breast (N=1,919)	983 (51.2%)	651 (33.9%)	285 (14.9%)	933 (48.6%)	153 (8.0%)	820 (42.7%)	54 (2.8%)	504 (26.3%)	116 (6.0%)	91 (4.7%)	320 (16.7%)
Lung (N=1,066)	60 (5.6%)	687 (64.4%)	319 (29.9%)	241 (22.6%)	51 (4.8%)	179 (16.8%)	12 (1.1%)	108 (10.1%)	38 (3.6%)	69 (6.5%)	196 (18.4%)
Colorectal (N=781)	20 (2.6%)	343 (43.9%)	418 (53.5%)	144 (18.4%)	60 (7.7%)	101 (12.9%)	8 (1.0%)	85 (10.9%)	45 (5.8%)	46 (5.9%)	131 (16.8%)
Prostate (N=252)	6 (2.4%)	205 (81.3%)	41 (16.3%)	49 (19.4%)	15 (6.0%)	37 (14.7%)	2 (0.8%)	23 (9.1%)	12 (4.8%)	12 (4.8%)	42 (16.7%)
Other (N=3,344)	348 (10.4%)	1,898 (56.8%)	1,098 (32.8%)	933 (27.9%)	293 (8.8%)	591 (17.7%)	65 (1.9%)	521 (15.6%)	212 (6.3%)	222 (6.6%)	632 (18.9%)

*CLL* Chronic lymphocytic leukemia, *NHL* Non-Hodgkin lymphoma

^1^Evaluated during the chemotherapy treatment period (the 30-day period prior to the episode index date)

^2^Regimens are classified as high-risk or intermediate-risk of FN based on the NCCN guidelines; all other regimens as low/indeterminate-risk. If an episode has a regimen that can be classified into ≥1 risk category, then the regimen will be classified by the highest risk regimen. Docetaxel and topotecan required diagnosis codes for specific cancers for categorization. Doxorubicin and cyclophosphamide required consideration of number and length of cycles over 180 days pre-index for categorization.

^3^Prophylactic use of CSF/antibiotic=prescription on the date of or within 4 days after the first chemotherapy administration.

^4^Use as treatment=prescription >4 days after the first chemotherapy administration

### Healthcare resource utilization and costs

#### All-cause utilization and costs

Of all episodes, 93.1% had ≥ 1 hospitalization, 14.9% had ≥ 1 ER visit, 87.8% had ≥ 1 outpatient physician office visit, 91.8% had ≥ 1 other outpatient claim, and 94.0% had ≥ 1 pharmacy claim. The mean number of healthcare visits (inpatient, ER, and outpatient) per episode was 24 visits (median of 17) across all episodes. Most visits were classified as other outpatient visits (such as non-physician; mean of 20 per episode). By comorbidity score category, HCRU increased with increasing comorbid burden: NCI score category=0 had a mean of 23.2 total all-cause visits; NCI score category >0-2, 24.7 visits; NCI score category ≥2, 25.0 visits. The mean total all-cause costs per episode was $35,899, driven by hospitalization costs ($32,704) and non-physician office outpatient costs ($4,913). For episodes with hospitalization, the mean length of stay (LOS) was 7.8 days; however, these episodes had higher financial burden than those without hospitalizations ($37,968 and $14,574, respectively) due to inpatient costs. Mean total costs increased with increase in NCI score (category=0, $33,813; NCI score category >0-2, $36,597; NCI score category ≥2, $37,713).

#### FN-related utilization and costs

All episodes had ≥1 FN-related healthcare visit; and 92.8% of episodes had ≥1 FN-related hospitalization during the episode period. The mean total FN-related costs per episode was $25,176; driven by hospitalization ($26,262) but also by GCSF treatment ($6,240). Episodes with ≥1 FN-related hospitalization had a mean hospital LOS of 6.9 days and had higher costs than episodes without a FN-related hospitalization ($26,868 and $7,738, respectively). When assessing FN-related treatments during the episode, 16.7% had ≥1 long-acting GCSF claim, 8.4% had ≥1 short-acting GCSF claim, and 40.3% had ≥1 incident antimicrobial claim. Mean costs for GCSF and antimicrobial pharmacy claims during the episode period was $6,240 and $229, respectively.

See Table 5 for all utilization and cost results.

**Table 5:** All-cause and FN-related healthcare utilization and costs during the FN episode, overall and sub-group

	All-Cause				FN-Related^1^						
	Total	Inpatient	Emergency Room	Physician	Pharmacy	Total	Inpatient	Emergency Room	Physician	GCSF	Anti-microbial
All Episodes (*N*=7,033) (*N*,%)	7,033 (100.0%)	6,544 (93.0%)	1,048 (14.9%)	6,178 (87.8%)	6,613 (94.0%)	7,033 (100.0%)	6,527 (92.8%)	675 (9.6%)	2,642 (37.6%)	1,174 (16.7%)	2,834 (40.3%)
Number of visits/IP LOS (mean, SD)	24.2 (23.1)	7.8 (8.5)	1.3 (0.8)	4.8 (6.5)	11.6 (8.3)	5.1 (9.3)	6.9 (7.2)	1.2 (0.5)	3.1 (4.5)	590 (8.4)	2.2 (2.1)
Cost^2^ (mean, SD)	$35,899 ($52,183)	$32,704 ($50,140)	$1,940 ($2,908)	$798 ($2,281)	$6,837 ($10,942)	$25,176 ($39,943)	$26,262 ($40,703)	$1,770 ($2,528)	$510 ($1,454)	$6,240 ($5,183)	$229 ($1,131)
Episode Type (N, %)											
With hospitalization (*N*=6,411)	6,411 (100.0%)	6,411 (100.0%)	597 (9.3%)	5,581 (87.1%)	5,993 (93.5%)	6,411 (100.0%)	6,411 (100.0%)	232 (3.6%)	2,267 (35.4%)	1,057 (16.5%)	2,459 (38.4%)
Number of visits (mean, SD)	23.1 (22.7)	7.8 (8.5)	1.4 (0.8)	4.8 (6.5)	11.4 (8.2)	4.1 (7.8)	7.0 (7.2)	1.1 (0.5)	3.1 (4.5)	476 (7.4)	2.0 (2.0)
Cost^2^ (mean, SD)	$37,968 ($53,814)	$32,778 ($50,481)	$1,601 ($2,772)	$793 ($2,359)	$6,756 ($10,921)	$26,868 ($41,183)	$26,354 ($40,910)	$902 ($1,592)	$489 ($1,498)	$6,363 ($5,180)	$227 ($1,157)
Without hospitalization (*N*=622)	622 (100.0%)	133 (21.4%)	451 (72.5%)	597 (96.0%)	620 (99.7%)	622 (100.0%)	116 (18.6%)	443 (71.2%)	375 (60.3%)	117 (18.8%)	375 (60.3%)
Number of visits (mean, SD)	35.6 (24.8)	7.2 (7.9)	1.3 (0.7)	4.9 (6.0)	13.4 (9.3)	15.6 (15.3)	6.0 (5.4)	1.2 (0.6)	3.2 (4.3)	114 (18.3)	3.2 (2.5)
Cost^2^ (mean, SD)	$14,574 ($21,060)	$29,140 ($29,292)	$2,380 ($3,023)	$841 ($1,363)	$7,626 ($11,119)	$7,738 ($15,030)	$21,136 ($26,517)	$2,203 ($2,785)	$641 ($1,142)	$5,363 ($5,131)	$238 ($940)

*IP* Inpatient, *LOS* Length of stay for the inpatient encounters

^1^FN-related is defined as a medical claim with a diagnosis code for neutropenia, infection, or fever during the episode, or a claim for an antimicrobial or GCSF administered/prescribed during the episode

^2^Costs converted to 2021 US dollars ($USD) using the Medical Component of the Consumer Pricing Index, and were calculated among patients that had at least one claim in that category

## Discussion

From 2014 to 2021, 7,033 episodes of FN were identified in the IQVIA PharMetrics® Plus database, with 91% of FN episodes involving hospitalization. Mean total cost per FN episode was $35,899, driven by costs incurred during hospitalization ($32,704); costs for FN episodes increased with patient comorbidity burden (NCI CCI score =0, $33,813, NCI CCI score ≥2, $37,713). A reduction in the number of FN episodes requiring hospitalization has the potential to have a significant impact on overall FN episode costs.

RFs were commonly seen in this analysis, with 86% of episodes having a surgery and 24% of episodes with radiation therapy prior to episode start date. Further, the median age of all episodes was 58 years, approaching the threshold of another RF (≥65 years of age); 21% of episodes already qualified for this RF and that number may grow given the aging population in the US. Our analysis knowingly underestimates this finding, as the dataset contains commercially insured patients, and it is not representative of the US population over 65 years of age. Our findings are similar to recent findings by Aslam et al. in 2023, a retrospective study of non-myeloid cancer patients receiving intermediate FN risk chemotherapy in administrative claims, that found 98% of patients had ≥ 1 RF [3]. This study found most episodes had an RF while less than 30% of all episodes received GCSF prophylaxis, a disparity echoed in the 46% with GCSF treatment in Aslam et al [3]. The disparity in RF and GCSF use highlights the opportunity to improve uptake of GCSF in appropriate situations. While the NCCN guidelines state GCSF should be “considered” in patients treated with intermediate or high FN risk chemotherapy regimen with ≥1 RF, our analysis suggests that, along with chemotherapy regimen, accurate individual RF consideration is crucial to determine appropriate GCSF usage.

While FN risk for common chemotherapy regimens are known, it is notable that many regimens in clinical use have not been evaluated for FN risk and thus are not classified in NCCN guidelines [1, 12]. Of the regimens with identified FN risk in this study, 72% of the FN episodes were preceded by intermediate FN-risk chemotherapy regimens, and 28% of the episodes were preceded by high FN-risk regimens. Notably, a sizable proportion (31%) of episodes had pre-episode chemotherapy regimens without an NCCN-assigned FN risk; these were identified as having low/undefined risk in this study. If a significant portion of the undefined FN-risk therapies are determined to confer a relatively high risk of FN, this would indicate an opportunity to optimize GCSF prophylaxis and improve population-based outcomes. Future research should focus on more precisely quantifying the FN-risk of various chemotherapy regimens, especially those that include newer therapies.

These cost estimates provide an update to the economic burden in FN episodes, as prior literature proxied costs from hospital charges while this study used recent real-world cost data. Further, assessing at the episode level allowed for a more thorough understanding of individual episode determinants. Prior studies estimated the mean LOS was 11 days (median of 6); this study found a lower overall LOS with a mean of 8 days (median of 5). Despite this, the financial burden per FN hospitalization appears to have increased substantially since Kuderer et al. in 2006; the prior estimate was a mean of $19,110 (median of $8,376) while this updated study found a mean of $32,704 (median of $19,901) [4].

This study found, episodes with hospitalization incurred higher all-cause and FN-related costs, indicating significant financial burden overall as nearly every FN episode included a hospital stay. Our results align with Aslam et al, finding inpatient stays represented nearly all costs in FN-related episodes [3]. FN-related costs per episode were slightly lower in their analysis in comparison to this study; however, the Aslam study was a retrospective patient-based study, and included only patients that had utilization of intermediate-FN risk chemotherapy. Patients with hematologic malignancies received on average more GCSF claims per FN episode than those with solid tumor malignancies, likely driving their higher overall costs. Aslam et al noted that higher per patient per month costs were seen in patients who did not receive GCSF (3); GCSF treatment was not a stratification in this study but should be considered for future studies. This is of particular importance with the introduction of biosimilars, as they offer a greatly reduced price of historically expensive treatments. Future research should further investigate the cost-effectiveness of various GCSF prophylaxis strategies to reduce FN, given their decreasing costs and in context of our study that shows an increase in FN hospitalization costs.

For this study, identification of FN episodes and other existing clinical conditions relied on availability of diagnosis codes, which may not cover all clinical events and could lead to misclassification bias or underestimation of qualifying episodes. Evaluation of treatments was limited to outpatient physician-administered and self-administered treatments via outpatient pharmacy, and this might lead to underestimation of GCSF or antimicrobial treatments if they were utilized during hospitalization.

This study used a database known to be representative of the commercially insured US national population for patients under 65 years of age, thus study results may not be representative to those above 65 years of age, and may not be generalizable to the US national population.

## Conclusions

The cost per FN hospitalization has increased substantially relative to earlier estimates. Almost a third of FN episodes followed administration of a regimen with no known NCCN-assigned FN risk, highlighting the opportunity that currently exists to mitigate or prevent FN hospitalization through approaches such as prophylactic GCSF use. As the cost of treating FN episodes increase and the price of GCSF decreases, this study provides important contemporary cost inputs for cost effectiveness evaluations of optimal treatment approaches for FN, such as GCSF as primary prophylaxis.

### Supplementary information

ESM 1 (DOCX 42 kb)
