# Supplementary material for: Chemotherapy-induced febrile neutropenia (FN): healthcare resource utilization (HCRU) and costs in commercially insured patients in the US
Source: Support Care Cancer. 2024 May 23;32(6):373. doi: 10.1007/s00520-024-08492-5 (PMC11111559; doi:10.1007/s00520-024-08492-5)
Supplement: Supplementary file 1 — (DOCX 42 kb) [file 520_2024_8492_MOESM1_ESM.docx]

## SUPPLEMENTAL TABLES

## Supplemental Table 1a – Sample Selection Criteria

| Inclusion criteria: |
| --- |
| Febrile neutropenia (FN) episodes during the identification period. Two definitions were used to identify episodes with FN: (i) Episodes with ≥1 inpatient medical claim with ≥1 diagnosis code for neutropenia and ≥1 diagnosis code for fever or infection on the same date; (ii) Episodes with ≥1 outpatient medical claim with a ≥1 diagnosis code for neutropenia and ≥1 diagnosis code for fever or infection on the same date and ≥1 claim for NCCN-recommended antibiotic therapy on the same date. Definitions for neutropenia, fever, and infection were modeled after those published by Weycker et al. and can be found in Supplementary Table 1b below (1). NCCN-recommended antibiotics were identified by three definitions: (i) ≥1 HCPCS code for an administration of a parenteral antibiotic; (ii) ≥1 pharmacy claim for ciprofloxacin and and ≥1 pharmacy claim for amoxicillin/clavulanate or clindamycin on the same date; (iii) ≥1 pharmacy claim for levofloxacin or moxifloxacin. |
| Episodes with ≥1 pharmacy or medical procedure claim for ≥1 chemotherapy medication or biologic during the 30-day period prior to the episode index date; from among the following: alemtuzumab; altretamine; amivantamab; arsenic; asparaginase; atezolizumab; avelumab; azacitidine; bcg; belantamab; bendamustine; bexarotene; bleomycin; blinatumomab; brentuximab; busulfan; cabazitaxel; calaspargase; capecitabine; carboplatin; carmustine; cemiplimab; cetuximab; chlorambucil; cisplatin; cladribine; clofarabine; cyclophosphamide; cytarabine; dacarbazine; dactinomycin; daratumumab; daunorubicin; decitabine; decitabine-cedazuridine; dinutuximab; docetaxel; dostarlimab; doxorubicin; durvalumab; efgartigimod; elotuzumab; enfortumab; epirubicin; eribulin; etoposide; floxuridine; fludarabine; fluorouracil; gemcitabine; gemtuzumab; hydroxyurea; idarubicin; ifosfamide; inotuzumab; interferon; ipilimumab; irinotecan; isatuximab; isotretinoin; ixabepilone; lenalidomide; leucovorin; levoleucovorin; lomustine; loncastuximab; lurbinectedin; margetuximab; mechlorethamine; melphalan; mercaptopurine; methotrexate; methoxsalen; mitomycin; mitoxantrone; mogamulizumab; moxetumomab; naxitamab; necitumumab; nelarabine; nivolumab; obinutuzumab; ofatumumab; olaratumab; omacetaxine; oxaliplatin; paclitaxel; panitumumab; pegaspargase; peginterferon; pembrolizumab; pemetrexed; pentostatin; pertuzumab; polatuzumab; pomalidomide; porfimer; pralatrexate; procarbazine; rituximab; ropeginterferon; sacituzumab; siltuximab; streptozocin; tafasitamab; tebentafusp; temozolomide; teniposide; thalidomide; thioguanine; thiotepa; tisotumab; topotecan; trabectedin; trastuzumab; tretinoin; trifluridine-tipiracil; valrubicin; venetoclax; vinblastine; vincristine; vinorelbine |
| Episodes with continuous enrollment during the 180-day period prior to the episode index date (pre-episode period). |
| Exclusion criteria: |
| ≥1 diagnosis code for neutropenia, infection, or fever during the 30-day period prior to the episode index date. |
| ≥1 medical claims with a procedure code for bone marrow or stem cell transplant or CAR-T cell therapy (from the following: axicabtagene; brexucabtagene; ciltacabtagene; idecabtagene; lisocabtagene; tisagenlecleucel; additional procedure codes for CAR-T preparation or unspecified engineered allogenic/autologous CAR-T administration) during the 180-day pre-episode period. |
| ≥1 medical claim with a diagnosis code for acute lymphocytic leukemia, acute myeloid leukemia, chronic myeloid leukemia, or myelodysplastic syndromes during the 180-day pre-episode period. |
| ≥1 medical claim with a diagnosis code for COVID-19 infection during the 180-day pre-episode period or 60-day post-episode period. |
| Data quality issues (missing age, missing sex, female sex with prostate cancer) |

## Supplemental Table 1b. Codes Indicative of Neutropenia, Fever, or Infection

| Neutropenia: ICD-9: 288.0; ICD-10: D70.9 |
| --- |
| Fever: ICD-9: 780.6; ICD-10: R50.9 |
| Infection: ICD-9: 002.0, 002.2, 002.9, 003.2, 003.20, 004, 004.1, 004.3, 004.9, 008.01, 008.2, 008.45, 036.2, 036.3, 036.42, 036.82, 036.9, 038.3, 038.40, 038.43, 039.1, 040.1, 040.81, 040.89, 041.02, 041.1, 041.11, 041.2, 041.42, 041.49, 041.6, 041.7, 041.81, 041.83, 041.84, 041.86, 041.9, 101, 112.5, 112.84, 115.03, 115.19, 116.1, 117.6, 320, 320.1, 320.7, 324.0, 360.01, 380.14, 383.02, 461.1, 461.2, 475, 482, 482.32, 482.4, 482.40, 482.42, 482.81, 482.83, 482.89, 482.9, 494.0, 494.1, 510, 522.7, 528.3, 541, 562.13, 567, 567.0, 567.1, 567.2, 567.3, 567.89, 567.9, 569.5, 569.61, 590, 590.10, 590.2, 590.3, 601.2, 601.9, 680.0, 680.8, 680.9, 681, 682, 682.0, 682.5, 682.7, 682.9, 686.1, 711.02, 711.03, 711.04, 711.08, 711.09, 728.86, 730.01, 730.09, 730.1, 730.10, 730.12, 730.14, 730.15, 730.25, 730.26, 730.29, 730.38, 730.70, 730.78, 730.81, 730.85, 730.9, 730.98, 790.7, 995.91, 999.3, 999.31, 999.32, A01.0, A01.03, A02.2, A02.8, A02.9, A04, A04.0, A04.6, A04.7, A04.71, A38, A39.0, A39.4, A39.5, A39.81, A39.82, A40.8, A41.02, A41.3, A41.5, A41.53, A41.89, A42.82, A42.89, A42.9, A43.0, A43.8, A48.5, A49.01, A49.2, A49.8, A54.43, A69.1, B37.1, B37.81, B37.9, B38.7, B38.8, B38.89, B39, B39.2, 002.3, 003, 003.22, 003.29, 008.0, 008.1, 008.46, 008.49, 008.5, 034.1, 036.4, 036.40, 036.41, 036.81, 038, 038.0, 038.19, 039.0, 039.3, 039.4, 040.2, 040.41, 040.8, 041.04, 041.12, 041.19, 041.85, 112.4, 114.1, 114.2, 114.3, 115.01, 115.05, 115.10, 115.12, 115.13, 115.15, 115.91, 115.92, 115.94, 116.0, 116.2, 117.0, 117.1, 117.2, 117.5, 117.8, 117.9, 118, 320.3, 320.82, 320.89, 360.00, 360.03, 376.03, 376.04, 383.00, 421, 421.0, 421.9, 461.3, 461.8, 462, 482.41, 482.8, 485, 494, 510.9, 513, 513.0, 526.4, 540, 540.1, 540.9, 562.11, 567.38, 567.81, 590.00, 590.01, 590.11, 590.80, 590.9, 601.3, 675.1, 675.11, 675.12, 675.14, 680, 680.1, 680.4, 681.0, 681.11, 681.9, 682.1, 682.6, 682.8, 683, 686.0, 686.09, 686.8, 686.9, 711.00, 711.01, 711.07, 730.0, 730.02, 730.04, 730.05, 730.08, 730.11, 730.13, 730.18, 730.22, 730.24, 730.3, 730.31, 730.33, 730.39, 730.71, 730.74, 730.86, 730.88, 730.93, 730.94, 730.97, 785.4, 785.52, 995.92, 998.5, 998.51, 998.59, 999.39; ICD-10: A01, A01.00, A01.05, A01.1, A02, A02.1, A02.20, A02.23, A02.25, A03.0, A03.1, A03.8, A04.2, A04.3, A04.8, A38.0, A38.8, A38.9, A39, A39.1, A39.52, A39.8, A40.0, A40.1, A40.3, A41.01, A41.1, A41.2, A41.4, A41.59, A41.81, A42, A42.8, A42.81, A43.1, A46, 002, 002.1, 003.23, 003.24, 003.8, 004.8, 008.00, 008.02, 008.09, 008.3, 008.4, 008.41, 008.43, 035, 036, 036.0, 036.1, 036.43, 036.89, 038.1, 038.10, 038.11, 038.41, 038.49, 038.9, 039, 039.2, 039.8, 039.9, 040, 040.0, 040.42, 040.82, 041, 041.0, 041.03, 041.05, 041.09, 041.10, 041.43, 041.8, 041.82, 112.0, 112.82, 112.83, 112.85, 112.89, 114.0, 114.5, 114.9, 115, 115.0, 115.1, 115.11, 115.14, 115.9, 115.95, 115.99, 117.3, 117.4, 320.0, 320.81, 320.9, 324.1, 360.0, 376.0, 376.01, 376.02, 421.1, 461.9, 463, 481, 482.0, 482.3, 482.31, 482.39, 482.49, 491.21, 510.0, 527.3, 562.01, 562.03, 566, 567.21, 567.23, 567.31, 567.39, 567.82, 572.0, 575.0, 590.8, 590.81, 601.0, 675.13, 680.5, 681.02, 681.1, 682.2, 682.3, 682.4, 685.0, 686, 686.01, 730.07, 730.16, 730.19, 730.20, 730.21, 730.27, 730.28, 730.30, 730.34, 730.35, 730.36, 730.37, 730.7, 730.8, 730.82, 730.89, 730.90, 730.92, 730.96, 999.34, A01.02, A01.04, A01.09, A01.3, A01.4, A02.21, A02.29, A03, A03.9, A04.1, A04.9, A38.1, A39.2, A39.83, A39.84, A39.89, A40, A40.9, A41.0, A41.51, A42.1, A42.7, A43.9, A48.1, A48.4, A69.0, B37, B37.2, B37.41, B37.49, B39.1, B39.9, B40.2, B40.7, B42.1, B42.8, B42.89, B42.9, B47.0, B95.0, B95.3, 003.0, 003.1, 003.21, 003.9, 004.0, 004.2, 008.03, 008.04, 008.42, 008.44, 008.47, 034, 034.0, 036.8, 038.12, 038.2, 038.4, 038.42, 038.44, 038.8, 040.3, 040.4, 041.00, 041.01, 041.3, 041.4, 041.41, 041.5, 041.89, 112.8, 112.81, 114, 114.4, 115.00, 115.02, 115.04, 115.09, 115.90, 115.93, 116, 117, 117.7, 320.2, 320.8, 321.0, 321.1, 324, 324.9, 360.02, 360.04, 376.00, 383.0, 383.01, 420.99, 461, 461.0, 482.1, 482.2, 482.30, 482.82, 482.84, 486, 513.1, 522.5, 540.0, 542, 567.22, 567.29, 567.8, 590.0, 590.1, 599.0, 601, 601.1, 601.4, 601.8, 675.10, 680.2, 680.3, 680.6, 680.7, 681.00, 681.01, 681.10, 686.00, 711.0, 711.05, 711.06, 730, 730.00, 730.03, 730.06, 730.17, 730.2, 730.23, 730.32, 730.72, 730.73, 730.75, 730.76, 730.77, 730.79, 730.80, 730.83, 730.84, 730.87, 730.91, 730.95, 730.99, 999.33, A01.01, A01.2, A02.0, A02.22, A02.24, A03.2, A03.3, A04.4, A04.5, A04.72, A39.3, A39.50, A39.51, A39.53, A39.9, A41, A41.50, A41.52, A41.8, A41.9, A42.0, A42.2, A43, A48, A48.0, A48.52, A48.8, A49, A49.02, A49.1, A49.3, A49.9, B37.0, B37.6, B37.8, B37.83, B38, B38.1, B38.2, B38.3, B38.4, B39.5, B40.0, B40.8, B40.89, B42, B42.81, B95.1, B95.6, B95.62, B95.7, B95.8, B39.4, B40, B40.1, B40.9, B42.82, B45.1, B47.1, B48.8, B95, B95.5, B95.61, B96.0, B96.2, B96.21, B96.4, E08.52, E13.52, G00.9, G07, H05.01, H05.031, H05.039, H05.112, H05.121, H05.219, H05.221, H05.223, H05.229, H05.232, H05.243, H05.253, H05.259, H05.26, H05.261, H05.319, H05.321, H05.331, H05.332, H05.333, H05.34, H05.341, H05.349, H05.353, H05.401, H05.403, H05.421, H05.423, H05.50, H05.52, H05.812, H05.813, H05.819, H05.82, H44.001, H44.002, H44.011, H44.423, H60.21, H60.23, H70.002, H70.09, I33.0, I70.262, I70.361, I70.362, I70.468, I70.561, I70.562, I70.663, I70.668, I70.761, I70.763, I70.768, I70.769, J01, J01.00, J01.01, J01.10, J01.21, J01.31, J02.9, J03.0, J03.9, J03.91, J15.1, J15.212, J15.3, J18, J18.2, J20.2, J44.1, J47.1, J85, J85.2, K35, K36, K50.914, K57.01, K57.32, K57.40, K57.8, K57.80, K61.0, K61.1, K61.3, K61.5, K63.0, K65.1, K65.3, K65.8, K65.9, K68.11, K90.81, L02.03, L02.11, L02.213, L02.215, L02.22, L02.221, L02.223, L02.224, L02.225, L02.229, L02.236, L02.239, L02.3, L02.41, L02.419, L02.423, L02.432, L02.436, L02.5, L02.522, L02.53, L02.531, L02.532, L02.619, L02.621, L02.629, A48.2, A48.3, A48.51, A49.0, B37.3, B37.4, B37.42, B37.5, B37.7, B37.82, B37.84, B37.89, B38.0, B38.81, B38.9, B39.0, B39.3, B40.3, B40.81, B42.0, B42.7, B47, B47.9, B95.2, B95.4, B96.29, B96.3, B96.8, E09.52, E11.52, G00.1, G00.2, G00.8, G01, G02, G06, H05.012, H05.013, H05.021, H05.022, H05.033, H05.043, H05.1, H05.123, H05.21, H05.222, H05.231, H05.233, H05.241, H05.242, H05.25, H05.251, H05.252, H05.262, H05.3, H05.409, H05.412, H05.419, H05.429, H05.5, H05.51, H05.53, H05.8, H05.81, H05.821, H05.822, H44.01, H44.021, H44.029, H44.42, H70.009, H70.011, H70.013, I30.8, I70.26, I70.263, I70.268, I70.36, I70.469, I70.56, I70.76, J01.0, J01.20, J01.4, J01.80, J01.9, J02, J03.00, J03.80, J13, J14, J15, J15.4, J15.7, J15.9, J18.1, J18.8, J18.9, J20.0, J47, J47.0, J85.3, K11.3, K35.21, K35.32, K35.89, K50.114, K51.314, K57.00, K57.12, K57.13, K57.2, K57.21, K57.33, K61.31, K65.0, K65.4, L02.02, L02.1, L02.12, L02.2, L02.219, L02.222, L02.233, L02.234, L02.235, L02.412, L02.416, L02.429, L02.435, L02.521, L02.529, L02.61, L02.612, L02.62, L02.622, L02.631, L02.639, L02.818, L02.82, L02.83, L02.831, L03.012, B96, B96.1, B96.22, B96.6, B96.82, B96.89, G00, G00.3, G04.2, G06.1, H05, H05.0, H05.011, H05.019, H05.02, H05.023, H05.029, H05.03, H05.042, H05.10, H05.113, H05.129, H05.20, H05.211, H05.212, H05.22, H05.263, H05.30, H05.311, H05.313, H05.32, H05.339, H05.342, H05.35, H05.351, H05.411, H05.42, H05.811, H05.823, H05.9, H44.009, H44.019, H44.02, H44.022, H44.023, H44.422, H60.20, H70.00, H70.01, H70.091, I30.9, I33, I33.9, I70.261, I70.269, I70.363, I70.369, I70.461, I70.462, I70.66, I70.661, I70.662, I70.669, I70.762, J01.11, J01.2, J01.3, J01.30, J01.8, J01.81, J01.90, J02.8, J03.01, J03.90, J15.2, J20.1, J36, J47.9, J85.0, J86.0, K04.7, K35.3, K35.33, K35.80, K35.890, K35.891, K37, K50.814, K51.814, K57.4, K57.53, K57.93, K61.2, K61.39, K65, K94.12, L02, L02.01, L02.13, L02.211, L02.214, L02.226, L02.23, L02.232, L02.31, L02.32, L02.411, L02.413, L02.42, L02.421, L02.425, L02.433, L02.439, L02.512, L02.52, L02.539, L02.81, L02.811, L02.92, L03, L03.011, L03.029, L03.03, L03.039, L03.04, L03.041, L03.114, L03.119, L03.126, L03.129, L03.22, L03.221, L03.31, L03.311, L03.312, L03.314, L03.325, L03.811, L03.818, L03.89, L04.1, L04.2, B96.20, B96.23, B96.5, B96.7, B96.81, E10.52, G00.0, G06.0, G06.2, H05.00, H05.032, H05.04, H05.041, H05.049, H05.11, H05.111, H05.119, H05.12, H05.122, H05.2, H05.213, H05.23, H05.239, H05.24, H05.249, H05.269, H05.31, H05.312, H05.322, H05.323, H05.329, H05.33, H05.343, H05.352, H05.359, H05.4, H05.40, H05.402, H05.41, H05.413, H05.422, H05.829, H05.89, H44.00, H44.003, H44.012, H44.013, H44.421, H44.429, H60.2, H60.22, H70.001, H70.003, H70.012, H70.019, H70.092, H70.093, H70.099, I70.368, I70.46, I70.463, I70.563, I70.568, I70.569, I96, J01.1, J01.40, J01.41, J01.91, J02.0, J03, J03.8, J03.81, J15.0, J15.20, J15.21, J15.211, J15.29, J15.5, J15.6, J15.8, J18.0, J85.1, J86, J86.9, K04.6, K12.2, K35.2, K35.20, K35.30, K35.31, K35.8, K50.014, K51.014, K51.214, K51.414, K51.514, K51.914, K57.0, K57.20, K57.41, K57.52, K57.81, K57.92, K61, K61.4, K65.2, K75.0, K81.0, K94.02, L02.0, L02.21, L02.212, L02.216, L02.231, L02.33, L02.4, L02.414, L02.415, L02.422, L02.424, L02.426, L02.43, L02.431, L02.434, L02.51, L02.511, L02.519, L02.6, L02.611, L02.63, L02.632, L02.8, L02.821, L02.838, L02.91, L02.828, L02.9, L03.01, L03.019, L03.022, L03.042, L03.111, L03.113, L03.115, L03.21, L03.213, L03.3, L03.313, L03.321, L03.323, L03.324, L03.81, L03.898, L03.9, L04.3, L04.8, L05.01, L05.02, L08.0, M00.019, M00.022, M00.062, M00.08, M00.11, M00.112, M00.131, M00.151, M00.169, M00.18, M00.20, M00.211, M00.212, M00.219, M00.22, M00.222, M00.23, M00.239, M00.241, M00.251, M00.252, M00.26, M00.261, M00.269, M00.29, M00.81, M00.821, M00.83, M00.859, M00.86, M00.862, M00.872, M00.88, M46.2, M46.20, M46.23, M46.26, M46.27, M72.6, M86.0, M86.02, M86.021, M86.03, M86.039, M86.049, M86.05, M86.06, M86.062, M86.07, M86.119, M86.121, M86.132, M86.14, M86.142, M86.149, M86.159, M86.162, M86.169, M86.171, M86.212, M86.229, M86.231, M86.24, M86.249, M86.27, M86.271, M86.29, M86.3, M86.311, M86.329, M86.34, M86.342, M86.349, M86.35, M86.351, M86.36, M86.362, M86.379, M86.39, M86.411, M86.441, M86.442, M86.451, M86.46, M86.469, M86.48, M86.50, M86.511, M86.52, M86.531, M86.552, M86.579, M86.58, M86.632, M86.64, M86.65, M86.651, M86.671, M86.672, M86.68, M86.8X1, M86.8X5, N11.0, N15.0, N39.0, O91.1, O91.12, T80.219A, L03.021, L03.049, L03.1, L03.11, L03.12, L03.123, L03.125, L03.222, L03.315, L03.316, L03.319, L03.322, L03.326, L03.327, L03.329, L03.8, L04, L04.0, M00.0, M00.039, M00.04, M00.05, M00.052, M00.06, M00.079, M00.09, M00.10, M00.111, M00.12, M00.121, M00.141, M00.142, M00.15, M00.152, M00.16, M00.17, M00.171, M00.179, M00.232, M00.242, M00.249, M00.259, M00.27, M00.279, M00.8, M00.80, M00.811, M00.819, M00.82, M00.829, M00.839, M00.842, M00.851, M00.871, M46.22, M86, M86.00, M86.011, M86.019, M86.022, M86.029, M86.031, M86.032, M86.042, M86.079, M86.12, M86.13, M86.152, M86.16, M86.172, M86.179, M86.18, M86.221, M86.232, M86.239, M86.241, M86.251, M86.28, M86.341, M86.359, M86.369, M86.372, M86.40, M86.422, M86.431, M86.44, M86.45, M86.452, M86.459, M86.461, M86.472, M86.49, M86.512, M86.522, M86.539, M86.54, M86.542, M86.559, M86.562, M86.569, M86.571, M86.59, M86.611, M86.622, M86.631, M86.641, M86.652, M86.662, M86.8, M86.8X7, M86.8X8, M86.8X9, N11.8, N41.0, N41.1, N41.3, N41.9, O91.111, O91.119, T81.40XA, T81.44XA, T81.4XXA, T82.7XXA, T83.518A, T83.593A, T83.598A, L04.9, M00.01, M00.011, M00.012, M00.021, M00.029, M00.032, M00.059, M00.061, M00.07, M00.071, M00.122, M00.129, M00.139, M00.159, M00.161, M00.172, M00.19, M00.231, M00.24, M00.271, M00.84, M00.849, M00.87, M00.89, M00.9, M27.2, M46.21, M46.25, M46.28, M86.01, M86.012, M86.041, M86.059, M86.069, M86.072, M86.09, M86.1, M86.10, M86.111, M86.122, M86.139, M86.141, M86.15, M86.151, M86.17, M86.19, M86.20, M86.21, M86.211, M86.222, M86.23, M86.242, M86.252, M86.259, M86.262, M86.269, M86.272, M86.30, M86.312, M86.319, M86.32, M86.321, M86.33, M86.339, M86.352, M86.361, M86.38, M86.41, M86.412, M86.419, M86.421, M86.43, M86.432, M86.449, M86.5, M86.51, M86.519, M86.529, M86.53, M86.541, M86.549, M86.551, M86.56, M86.572, M86.60, M86.621, M86.629, M86.642, M86.649, M86.659, M86.66, M86.669, M86.69, M86.8X, M86.8X6, N11, N11.9, N12, N13.6, N15.1, N15.8, N15.9, N41, N41.4, N41.8, O91.112, R65.20, T80.29XA, T83.510A, T83.512A, T83.592A, T84.60XA, T84.610A, T84.613A, T84.615A, T84.619A, T84.621A, T84.622A, T84.625A, T85.71XA, L02.93, L03.0, L03.02, L03.031, L03.032, L03.112, L03.116, L03.121, L03.122, L03.124, L03.2, L03.211, L03.212, L03.317, L03.32, L03.891, L03.90, L03.91, M00, M00.00, M00.02, M00.03, M00.031, M00.041, M00.042, M00.049, M00.051, M00.069, M00.072, M00.1, M00.119, M00.13, M00.132, M00.14, M00.149, M00.162, M00.2, M00.21, M00.221, M00.229, M00.25, M00.262, M00.272, M00.28, M00.812, M00.822, M00.831, M00.832, M00.841, M00.85, M00.852, M00.861, M00.869, M00.879, M46.24, M86.04, M86.051, M86.052, M86.061, M86.071, M86.08, M86.11, M86.112, M86.129, M86.131, M86.161, M86.2, M86.219, M86.22, M86.25, M86.26, M86.261, M86.279, M86.31, M86.322, M86.331, M86.332, M86.37, M86.371, M86.4, M86.42, M86.429, M86.439, M86.462, M86.47, M86.471, M86.479, M86.521, M86.532, M86.55, M86.561, M86.57, M86.6, M86.61, M86.612, M86.619, M86.62, M86.63, M86.639, M86.661, M86.67, M86.679, M86.8X0, M86.8X2, M86.8X3, M86.8X4, M86.9, N10, N11.1, N15, N41.2, O91.11, O91.113, O91.13, R65.2, R65.21, T79.8XXA, T80.211A, T80.212A, T80.218A, T81.41XA, T81.49XA, T83.511A, T83.590A, T83.591A, T83.69XA, T84.612A, T84.623A, T80.22XA, T81.42XA, T81.43XA, T82.6XXA, T83.51XA, T83.59XA, T83.61XA, T83.6XXA, T84.51XA, T84.52XA, T84.629A, T85.731A, T85.79XA, T83.62XA, T84.50XA, T84.53XA, T84.54XA, T84.59XA, T84.611A, T84.614A, T84.620A, T84.624A, T85.72XA, T85.730A, T85.732A, T85.733A, T85.734A, T85.735A, T84.63XA, T84.69XA, T85.738A, T88.0XXA |

## Supplemental Table 2. Codes for Specific Malignancy Type Stratification

| Breast | ICD-9: 174, 174.0, 174.1, 174.2, 174.3, 174.4, 174.5, 174.6, 174.8, 174.9, 175, 175.0, 175.9; ICD-10: C50, C50.0, C50.01, C50.011, C50.012, C50.019, C50.02, C50.021, C50.022, C50.029, C50.1, C50.11, C50.111, C50.112, C50.119, C50.12, C50.121, C50.122, C50.129, C50.2, C50.21, C50.211, C50.212, C50.219, C50.22, C50.221, C50.222, C50.229, C50.3, C50.31, C50.311, C50.312, C50.319, C50.32, C50.321, C50.322, C50.329, C50.4, C50.41, C50.411, C50.412, C50.419, C50.42, C50.421, C50.422, C50.429, C50.5, C50.51, C50.511, C50.512, C50.519, C50.52, C50.521, C50.522, C50.529, C50.6, C50.61, C50.611, C50.612, C50.619, C50.62, C50.621, C50.622, C50.629, C50.8, C50.81, C50.811, C50.812, C50.819, C50.82, C50.821, C50.822, C50.829, C50.9, C50.91, C50.911, C50.912, C50.919, C50.92, C50.921, C50.922, C50.929 |
| --- | --- |
| Colorectal | ICD-9: 153, 153.0, 153.1, 153.2, 153.3, 153.4, 153.5, 153.6, 153.7, 153.8, 153.9, 154, 154.0, 154.1, 154.2, 154.8; ICD-10: C18, C18.0, C18.1, C18.2, C18.3, C18.4, C18.5, C18.6, C18.7, C18.8, C18.9, C19, C20, C21, C21.1, C21.8, C49.A5 |
| Lung | ICD-9: 162, 162.2, 162.3, 162.4, 162.5, 162.8, 162.9; ICD-10: C34, C34.0, C34.00, C34.01, C34.02, C34.1, C34.10, C34.11, C34.12, C34.2, C34.3, C34.30, C34.31, C34.32, C34.8, C34.80, C34.81, C34.82, C34.9, C34.90, C34.91, C34.92 |
| Prostate | ICD-9: 185; ICD-10: C61 |
| Other-solid | ICD-9: 140, 140.0, 140.1, 140.3, 140.4, 140.5, 140.6, 140.8, 140.9, 141, 141.0, 141.1, 141.2, 141.3, 141.4, 141.5, 141.6, 141.8, 141.9, 142, 142.0, 142.1, 142.2, 142.8, 142.9, 143, 143.0, 143.1, 143.8, 143.9, 144, 144.0, 144.1, 144.8, 144.9, 145, 145.0, 145.1, 145.2, 145.3, 145.4, 145.5, 145.6, 145.8, 145.9, 146, 146.0, 146.1, 146.2, 146.3, 146.4, 146.5, 146.6, 146.7, 146.8, 146.9, 147, 147.0, 147.1, 147.2, 147.3, 147.8, 147.9, 148, 148.0, 148.1, 148.2, 148.3, 148.8, 148.9, 149, 149.0, 149.1, 149.8, 149.9, 150, 150.0, 150.1, 150.2, 150.3, 150.4, 150.5, 150.8, 150.9, 151, 151.0, 151.1, 151.2, 151.3, 151.4, 151.5, 151.6, 151.8, 151.9, 152, 152.0, 152.1, 152.2, 152.3, 152.8, 152.9, 154.3, 155, 155.0, 155.1, 155.2, 156, 156.0, 156.1, 156.2, 156.8, 156.9, 157, 157.0, 157.1, 157.2, 157.3, 157.4, 157.8, 157.9, 158, 158.0, 158.8, 158.9, 159, 159.0, 159.1, 159.8, 159.9, 160, 160.0, 160.1, 160.2, 160.3, 160.4, 160.5, 160.8, 160.9, 161, 161.0, 161.1, 161.2, 161.3, 161.8, 161.9, 162.0, 163, 163.0, 163.1, 163.8, 163.9, 164, 164.0, 164.1, 164.2, 164.3, 164.8, 164.9, 165, 165.0, 165.8, 165.9, 170, 170.0, 170.1, 170.2, 170.3, 170.4, 170.5, 170.6, 170.7, 170.8, 170.9, 171, 171.0, 171.2, 171.3, 171.4, 171.5, 171.6, 171.7, 171.8, 171.9, 172, 172.0, 172.1, 172.2, 172.3, 172.4, 172.5, 172.6, 172.7, 172.8, 172.9, 173, 173.0, 173.00, 173.01, 173.02, 173.09, 173.1, 173.10, 173.11, 173.12, 173.19, 173.2, 173.20, 173.21, 173.22, 173.29, 173.3, 173.30, 173.31, 173.32, 173.39, 173.4, 173.40, 173.41, 173.42, 173.49, 173.5, 173.50, 173.51, 173.52, 173.59, 173.6, 173.60, 173.61, 173.62, 173.69, 173.7, 173.70, 173.71, 173.72, 173.79, 173.8, 173.80, 173.81, 173.82, 173.89, 173.9, 173.90, 173.91, 173.92, 173.99, 176, 176.0, 176.1, 176.2, 176.3, 176.4, 176.5, 176.8, 176.9, 179, 180, 180.0, 180.1, 180.8, 180.9, 181, 182, 182.0, 182.1, 182.8, 183, 183.0, 183.2, 183.3, 183.4, 183.5, 183.8, 183.9, 184, 184.0, 184.1, 184.2, 184.3, 184.4, 184.8, 184.9, 186, 186.0, 186.9, 187, 187.1, 187.2, 187.3, 187.4, 187.5, 187.6, 187.7, 187.8, 187.9, 188, 188.0, 188.1, 188.2, 188.3, 188.4, 188.5, 188.6, 188.7, 188.8, 188.9, 189, 189.0, 189.1, 189.2, 189.3, 189.4, 189.8, 189.9, 190, 190.0, 190.1, 190.2, 190.3, 190.4, 190.5, 190.6, 190.7, 190.8, 190.9, 191, 191.0, 191.1, 191.2, 191.3, 191.4, 191.5, 191.6, 191.7, 191.8, 191.9, 192, 192.0, 192.1, 192.2, 192.3, 192.8, 192.9, 193, 194, 194.0, 194.1, 194.3, 194.4, 194.5, 194.6, 194.8, 194.9, 209, 209.0, 209.00, 209.01, 209.02, 209.03, 209.1, 209.10, 209.11, 209.12, 209.13, 209.14, 209.15, 209.16, 209.17, 209.2, 209.20, 209.21, 209.22, 209.23, 209.24, 209.25, 209.26, 209.27, 209.29, 209.3, 209.30, 209.31, 209.32, 209.33, 209.34, 209.35, 209.36, 209.7, 209.70, 209.71, 209.72, 209.73, 209.74, 209.75, 209.79; ICD-10: C00, C00.0, C00.1, C00.2, C00.3, C00.4, C00.5, C00.6, C00.8, C00.9, C01, C02, C02.0, C02.1, C02.2, C02.3, C02.4, C02.8, C02.9, C03, C03.0, C03.1, C03.9, C04, C04.0, C04.1, C04.8, C04.9, C05, C05.0, C05.1, C05.2, C05.8, C05.9, C06, C06.0, C06.1, C06.2, C06.8, C06.80, C06.89, C06.9, C07, C08, C08.0, C08.1, C08.9, C09, C09.0, C09.1, C09.8, C09.9, C10, C10.0, C10.1, C10.2, C10.3, C10.4, C10.8, C10.9, C11, C11.0, C11.1, C11.2, C11.3, C11.8, C11.9, C12, C13, C13.0, C13.1, C13.2, C13.8, C13.9, C14, C14.0, C14.2, C14.8, C15, C15.3, C15.4, C15.5, C15.8, C15.9, C16, C16.0, C16.1, C16.2, C16.3, C16.4, C16.5, C16.6, C16.8, C16.9, C17, C17.0, C17.1, C17.2, C17.3, C17.8, C17.9, C21.0, C21.2, C22, C22.0, C22.1, C22.2, C22.3, C22.4, C22.7, C22.8, C22.9, C23, C24, C24.0, C24.1, C24.8, C24.9, C25, C25.0, C25.1, C25.2, C25.3, C25.4, C25.7, C25.8, C25.9, C26, C26.0, C26.1, C26.9, C30, C30.0, C30.1, C31, C31.0, C31.1, C31.2, C31.3, C31.8, C31.9, C32, C32.0, C32.1, C32.2, C32.3, C32.8, C32.9, C33, C37, C38, C38.0, C38.1, C38.2, C38.3, C38.4, C38.8, C39, C39.0, C39.9, C40, C40.0, C40.00, C40.01, C40.02, C40.1, C40.10, C40.11, C40.12, C40.2, C40.20, C40.21, C40.22, C40.3, C40.30, C40.31, C40.32, C40.8, C40.80, C40.81, C40.82, C40.9, C40.90, C40.91, C40.92, C41, C41.0, C41.1, C41.2, C41.3, C41.4, C41.9, C43, C43.0, C43.1, C43.10, C43.11, C43.111, C43.112, C43.12, C43.121, C43.122, C43.2, C43.20, C43.21, C43.22, C43.3, C43.30, C43.31, C43.39, C43.4, C43.5, C43.51, C43.52, C43.59, C43.6, C43.60, C43.61, C43.62, C43.7, C43.70, C43.71, C43.72, C43.8, C43.9, C44, C44.0, C44.00, C44.01, C44.02, C44.09, C44.1, C44.10, C44.101, C44.102, C44.1021, C44.1022, C44.109, C44.1091, C44.1092, C44.11, C44.111, C44.112, C44.1121, C44.1122, C44.119, C44.1191, C44.1192, C44.12, C44.121, C44.122, C44.1221, C44.1222, C44.129, C44.1291, C44.1292, C44.13, C44.131, C44.132, C44.1321, C44.1322, C44.139, C44.1391, C44.1392, C44.19, C44.191, C44.192, C44.1921, C44.1922, C44.199, C44.1991, C44.1992, C44.2, C44.20, C44.201, C44.202, C44.209, C44.21, C44.211, C44.212, C44.219, C44.22, C44.221, C44.222, C44.229, C44.29, C44.291, C44.292, C44.299, C44.3, C44.30, C44.300, C44.301, C44.309, C44.31, C44.310, C44.311, C44.319, C44.32, C44.320, C44.321, C44.329, C44.39, C44.390, C44.391, C44.399, C44.4, C44.40, C44.41, C44.42, C44.49, C44.5, C44.50, C44.500, C44.501, C44.509, C44.51, C44.510, C44.511, C44.519, C44.52, C44.520, C44.521, C44.529, C44.59, C44.590, C44.591, C44.599, C44.6, C44.60, C44.601, C44.602, C44.609, C44.61, C44.611, C44.612, C44.619, C44.62, C44.621, C44.622, C44.629, C44.69, C44.691, C44.692, C44.699, C44.7, C44.70, C44.701, C44.702, C44.709, C44.71, C44.711, C44.712, C44.719, C44.72, C44.721, C44.722, C44.729, C44.79, C44.791, C44.792, C44.799, C44.8, C44.80, C44.81, C44.82, C44.89, C44.9, C44.90, C44.91, C44.92, C44.99, C45, C45.0, C45.1, C45.2, C45.7, C45.9, C46, C46.0, C46.1, C46.2, C46.3, C46.4, C46.5, C46.50, C46.51, C46.52, C46.7, C46.9, C47, C47.0, C47.1, C47.10, C47.11, C47.12, C47.2, C47.20, C47.21, C47.22, C47.3, C47.4, C47.5, C47.6, C47.8, C47.9, C48, C48.0, C48.1, C48.2, C48.8, C49, C49.0, C49.1, C49.10, C49.11, C49.12, C49.2, C49.20, C49.21, C49.22, C49.3, C49.4, C49.5, C49.6, C49.8, C49.9, C49.A, C49.A0, C49.A1, C49.A2, C49.A3, C49.A4, C49.A9, C4A, C4A.0, C4A.1, C4A.10, C4A.11, C4A.111, C4A.112, C4A.12, C4A.121, C4A.122, C4A.2, C4A.20, C4A.21, C4A.22, C4A.3, C4A.30, C4A.31, C4A.39, C4A.4, C4A.5, C4A.51, C4A.52, C4A.59, C4A.6, C4A.60, C4A.61, C4A.62, C4A.7, C4A.70, C4A.71, C4A.72, C4A.8, C4A.9, C51, C51.0, C51.1, C51.2, C51.8, C51.9, C52, C53, C53.0, C53.1, C53.8, C53.9, C54, C54.0, C54.1, C54.2, C54.3, C54.8, C54.9, C55, C56, C56.1, C56.2, C56.3, C56.9, C57, C57.0, C57.00, C57.01, C57.02, C57.1, C57.10, C57.11, C57.12, C57.2, C57.20, C57.21, C57.22, C57.3, C57.4, C57.7, C57.8, C57.9, C58, C60, C60.0, C60.1, C60.2, C60.8, C60.9, C62, C62.0, C62.00, C62.01, C62.02, C62.1, C62.10, C62.11, C62.12, C62.9, C62.90, C62.91, C62.92, C63, C63.0, C63.00, C63.01, C63.02, C63.1, C63.10, C63.11, C63.12, C63.2, C63.7, C63.8, C63.9, C64, C64.1, C64.2, C64.9, C65, C65.1, C65.2, C65.9, C66, C66.1, C66.2, C66.9, C67, C67.0, C67.1, C67.2, C67.3, C67.4, C67.5, C67.6, C67.7, C67.8, C67.9, C68, C68.0, C68.1, C68.8, C68.9, C69, C69.0, C69.00, C69.01, C69.02, C69.1, C69.10, C69.11, C69.12, C69.2, C69.20, C69.21, C69.22, C69.3, C69.30, C69.31, C69.32, C69.4, C69.40, C69.41, C69.42, C69.5, C69.50, C69.51, C69.52, C69.6, C69.60, C69.61, C69.62, C69.8, C69.80, C69.81, C69.82, C69.9, C69.90, C69.91, C69.92, C70, C70.0, C70.1, C70.9, C71, C71.0, C71.1, C71.2, C71.3, C71.4, C71.5, C71.6, C71.7, C71.8, C71.9, C72, C72.0, C72.1, C72.2, C72.20, C72.21, C72.22, C72.3, C72.30, C72.31, C72.32, C72.4, C72.40, C72.41, C72.42, C72.5, C72.50, C72.59, C72.9, C73, C74, C74.0, C74.00, C74.01, C74.02, C74.1, C74.10, C74.11, C74.12, C74.9, C74.90, C74.91, C74.92, C75, C75.0, C75.1, C75.2, C75.3, C75.4, C75.5, C75.8, C75.9, C7A, C7A.0, C7A.00, C7A.01, C7A.010, C7A.011, C7A.012, C7A.019, C7A.02, C7A.020, C7A.021, C7A.022, C7A.023, C7A.024, C7A.025, C7A.026, C7A.029, C7A.09, C7A.090, C7A.091, C7A.092, C7A.093, C7A.094, C7A.095, C7A.096, C7A.098, C7A.1, C7A.8, C7B, C7B.0, C7B.00, C7B.01, C7B.02, C7B.03, C7B.04, C7B.09, C7B.1, C7B.8 |
| CLL/SLL | ICD-10: C83.0, C83.00, C83.01, C83.02, C83.03, C83.04, C83.05, C83.06, C83.07, C83.08, C83.09, C91.1, C91.10, C91.11, C91.12 |
| NHL | ICD-9: 200.2, 200.20, 200.21, 200.22, 200.23, 200.24, 200.25, 200.26, 200.27, 200.28, 200.3, 200.30, 200.31, 200.32, 200.33, 200.34, 200.35, 200.36, 200.37, 200.38, 200.4, 200.40, 200.41, 200.42, 200.43, 200.44, 200.45, 200.46, 200.47, 200.48, 200.6, 200.60, 200.61, 200.62, 200.63, 200.64, 200.65, 200.66, 200.67, 200.68, 200.7, 200.70, 200.71, 200.72, 200.73, 200.74, 200.75, 200.76, 200.77, 200.78, 200.8, 200.80, 200.81, 200.82, 200.83, 200.84, 200.85, 200.86, 200.87, 200.88, 202.0, 202.00, 202.01, 202.02, 202.03, 202.04, 202.05, 202.06, 202.07, 202.08, 202.1, 202.10, 202.11, 202.12, 202.13, 202.14, 202.15, 202.16, 202.17, 202.18, 202.2, 202.20, 202.21, 202.22, 202.23, 202.24, 202.25, 202.26, 202.27, 202.28, 202.7, 202.70, 202.71, 202.72, 202.73, 202.74, 202.75, 202.76, 202.77, 202.78, 202.8, 202.80, 202.81, 202.82, 202.83, 202.84, 202.85, 202.86, 202.87, 202.88; ICD_10: C82, C82.0, C82.00, C82.01, C82.02, C82.03, C82.04, C82.05, C82.06, C82.07, C82.08, C82.09, C82.1, C82.10, C82.11, C82.12, C82.13, C82.14, C82.15, C82.16, C82.17, C82.18, C82.19, C82.2, C82.20, C82.21, C82.22, C82.23, C82.24, C82.25, C82.26, C82.27, C82.28, C82.29, C82.3, C82.30, C82.31, C82.32, C82.33, C82.34, C82.35, C82.36, C82.37, C82.38, C82.39, C82.4, C82.40, C82.41, C82.42, C82.43, C82.44, C82.45, C82.46, C82.47, C82.48, C82.49, C82.5, C82.50, C82.51, C82.52, C82.53, C82.54, C82.55, C82.56, C82.57, C82.58, C82.59, C82.6, C82.60, C82.61, C82.62, C82.63, C82.64, C82.65, C82.66, C82.67, C82.68, C82.69, C82.8, C82.80, C82.81, C82.82, C82.83, C82.84, C82.85, C82.86, C82.87, C82.88, C82.89, C82.9, C82.90, C82.91, C82.92, C82.93, C82.94, C82.95, C82.96, C82.97, C82.98, C82.99, C83, C83.1, C83.10, C83.11, C83.12, C83.13, C83.14, C83.15, C83.16, C83.17, C83.18, C83.19, C83.3, C83.30, C83.31, C83.32, C83.33, C83.34, C83.35, C83.36, C83.37, C83.38, C83.39, C83.5, C83.50, C83.51, C83.52, C83.53, C83.54, C83.55, C83.56, C83.57, C83.58, C83.59, C83.7, C83.70, C83.71, C83.72, C83.73, C83.74, C83.75, C83.76, C83.77, C83.78, C83.79, C83.8, C83.80, C83.81, C83.82, C83.83, C83.84, C83.85, C83.86, C83.87, C83.88, C83.89, C83.9, C83.90, C83.91, C83.92, C83.93, C83.94, C83.95, C83.96, C83.97, C83.98, C83.99, C84, C84.0, C84.00, C84.01, C84.02, C84.03, C84.04, C84.05, C84.06, C84.07, C84.08, C84.09, C84.1, C84.10, C84.11, C84.12, C84.13, C84.14, C84.15, C84.16, C84.17, C84.18, C84.19, C84.4, C84.40, C84.41, C84.42, C84.43, C84.44, C84.45, C84.46, C84.47, C84.48, C84.49, C84.6, C84.60, C84.61, C84.62, C84.63, C84.64, C84.65, C84.66, C84.67, C84.68, C84.69, C84.7, C84.70, C84.71, C84.72, C84.73, C84.74, C84.75, C84.76, C84.77, C84.78, C84.79, C84.7A, C84.9, C84.90, C84.91, C84.92, C84.93, C84.94, C84.95, C84.96, C84.97, C84.98, C84.99, C84.A, C84.A0, C84.A1, C84.A2, C84.A3, C84.A4, C84.A5, C84.A6, C84.A7, C84.A8, C84.A9, C84.Z, C84.Z0, C84.Z1, C84.Z2, C84.Z3, C84.Z4, C84.Z5, C84.Z6, C84.Z7, C84.Z8, C84.Z9, C85, C85.1, C85.10, C85.11, C85.12, C85.13, C85.14, C85.15, C85.16, C85.17, C85.18, C85.19, C85.2, C85.20, C85.21, C85.22, C85.23, C85.24, C85.25, C85.26, C85.27, C85.28, C85.29, C85.8, C85.80, C85.81, C85.82, C85.83, C85.84, C85.85, C85.86, C85.87, C85.88, C85.89, C85.9, C85.90, C85.91, C85.92, C85.93, C85.94, C85.95, C85.96, C85.97, C85.98, C85.99, C86, C86.0, C86.1, C86.2, C86.3, C86.4, C86.5, C86.6, C88, C88.0, C88.2, C88.3, C88.4, C88.8, C88.9 |
| Other-hematologic | ICD-9: 200, 200.0, 200.00, 200.01, 200.02, 200.03, 200.04, 200.05, 200.06, 200.07, 200.08, 200.1, 200.10, 200.11, 200.12, 200.13, 200.14, 200.15, 200.16, 200.17, 200.18, 200.5, 200.50, 200.51, 200.52, 200.53, 200.54, 200.55, 200.56, 200.57, 200.58, 201, 201.0, 201.00, 201.01, 201.02, 201.03, 201.04, 201.05, 201.06, 201.07, 201.08, 201.1, 201.10, 201.11, 201.12, 201.13, 201.14, 201.15, 201.16, 201.17, 201.18, 201.2, 201.20, 201.21, 201.22, 201.23, 201.24, 201.25, 201.26, 201.27, 201.28, 201.4, 201.40, 201.41, 201.42, 201.43, 201.44, 201.45, 201.46, 201.47, 201.48, 201.5, 201.50, 201.51, 201.52, 201.53, 201.54, 201.55, 201.56, 201.57, 201.58, 201.6, 201.60, 201.61, 201.62, 201.63, 201.64, 201.65, 201.66, 201.67, 201.68, 201.7, 201.70, 201.71, 201.72, 201.73, 201.74, 201.75, 201.76, 201.77, 201.78, 201.9, 201.90, 201.91, 201.92, 201.93, 201.94, 201.95, 201.96, 201.97, 201.98, 202, 202.3, 202.30, 202.31, 202.32, 202.33, 202.34, 202.35, 202.36, 202.37, 202.38, 202.4, 202.40, 202.5, 202.50, 202.51, 202.52, 202.53, 202.54, 202.55, 202.56, 202.57, 202.58, 202.6, 202.60, 202.61, 202.62, 202.63, 202.64, 202.65, 202.66, 202.67, 202.68, 202.9, 202.90, 202.91, 202.92, 202.93, 202.94, 202.95, 202.96, 202.97, 202.98, 203, 203.0, 203.00, 203.01, 203.02, 204.1, 204.10, 204.11, 204.12; ICD-10: C81, C81.0, C81.00, C81.01, C81.02, C81.03, C81.04, C81.05, C81.06, C81.07, C81.08, C81.09, C81.1, C81.10, C81.11, C81.12, C81.13, C81.14, C81.15, C81.16, C81.17, C81.18, C81.19, C81.2, C81.20, C81.21, C81.22, C81.23, C81.24, C81.25, C81.26, C81.27, C81.28, C81.29, C81.3, C81.30, C81.31, C81.32, C81.33, C81.34, C81.35, C81.36, C81.37, C81.38, C81.39, C81.4, C81.40, C81.41, C81.42, C81.43, C81.44, C81.45, C81.46, C81.47, C81.48, C81.49, C81.7, C81.70, C81.71, C81.72, C81.73, C81.74, C81.75, C81.76, C81.77, C81.78, C81.79, C81.9, C81.90, C81.91, C81.92, C81.93, C81.94, C81.95, C81.96, C81.97, C81.98, C81.99, C90, C90.0, C90.00, C90.01, C90.02, C90.1, C90.10, C90.11, C90.12, C90.2, C90.20, C90.21, C90.22, C90.3, C90.30, C90.31, C90.32, C91 |

## Supplemental Table 3. Baseline Demographics by Additional Sub-groups

Table S3.1. Baseline demographic characteristics, by hematologic malignancy subtypes

| Demographic characteristics | | NHL  (N=1,010) | CLL  (N=148) | Other hematologic  (N=504) |
| --- | --- | --- | --- | --- |
| Age, years^3^ | Mean (SD) | 55.9 (15.7) | 61.6 (11.4) | 53.4 (17.1) |
|  | Median | 59 | 62 | 58 |
| Age category, years, n (%) | <18 | 24 (2.4%) | 0 (0.0%) | 19 (3.8%) |
|  | 18 to 59 | 505 (50.0%) | 53 (35.8%) | 260 (51.6%) |
|  | 60 to 74 | 385 (38.1%) | 74 (50.0%) | 193 (38.3%) |
|  | 75+ | 96 (9.5%) | 21 (14.2%) | 32 (6.3%) |
| Sex, n (%) | Male | 565 (55.9%) | 90 (60.8%) | 278 (55.2%) |
|  | Female | 445 (44.1%) | 58 (39.2%) | 226 (44.8%) |
| Payer type, n (%) | Commercial | 852 (84.4%) | 118 (79.7%) | 444 (88.1%) |
|  | Medicare | 92 (9.1%) | 21 (14.2%) | 36 (7.1%) |
|  | Medicaid | 21 (2.1%) | 3 (2.0%) | 7 (1.4%) |
|  | Unknown | 45 (4.5%) | 6 (4.1%) | 17 (3.4%) |
| Geographic region, n (%) | Northeast | 217 (21.5%) | 32 (21.6%) | 95 (18.8%) |
|  | Midwest | 284 (28.1%) | 38 (25.7%) | 132 (26.2%) |
|  | South | 342 (33.9%) | 55 (37.2%) | 193 (38.3%) |
|  | West | 162 (16.0%) | 23 (15.5%) | 80 (15.9%) |
|  | Unknown | 5 (0.5%) | 0 (0.0%) | 4 (0.8%) |
| Episode length in days | Mean (SD) | 31.1 (21.3) | 33.9 (21.4) | 29.0 (22.0) |
|  | Median (Min, Max) | 21 (1, 60) | 24 (2, 60) | 19 (1, 60) |

Abbreviations: CLL=chronic lymphocytic leukemia; NHL=Non-Hodgkin’s lymphoma; SD=standard deviation

Table S3.2. Baseline demographic characteristics, by solid tumor subtypes

| Demographic characteristics | | Breast (N=1,919) | Lung (N=1,066) | Colorectal (N=781) | Prostate (N=252) | Other solid tumor (N=3,344) |
| --- | --- | --- | --- | --- | --- | --- |
| Age, years | Mean (SD) | 55.1 (10.6) | 61.7 (10.3) | 57.7 (11.1) | 65.5 (11.0) | 53.9 (17.0) |
|  | Median | 56 | 62 | 58 | 64 | 59 |
| Age category, years, n (%) | <18 | 1 (0.1%) | 6 (0.6%) | 1 (0.1%) | 1 (0.4%) | 202 (6.0%) |
|  | 18 to 59 | 1,206 (62.8%) | 368 (34.5%) | 424 (54.3%) | 51 (20.2%) | 1,607 (48.1%) |
|  | 60 to 74 | 659 (34.3%) | 589 (55.3%) | 298 (38.2%) | 147 (58.3%) | 1,356 (40.6%) |
|  | 75+ | 53 (2.8%) | 103 (9.7%) | 58 (7.4%) | 53 (21.0%) | 179 (5.4%) |
| Sex, n (%) | Male | 12 (0.6%) | 534 (50.1%) | 375 (48.0%) | 252 (100.0%) | 1,570 (46.9%) |
|  | Female | 1,907 (99.4%) | 532 (49.9%) | 406 (52.0%) | 0 (0.0%) | 1,774 (53.1%) |
| Payer type, n (%) | Commercial | 1,754 (91.4%) | 860 (80.7%) | 688 (88.1%) | 191 (75.8%) | 2,943 (88.0%) |
|  | Medicare | 91 (4.7%) | 130 (12.2%) | 56 (7.2%) | 32 (12.7%) | 239 (7.1%) |
|  | Medicaid | 40 (2.1%) | 37 (3.5%) | 12 (1.5%) | 2 (0.8%) | 67 (2.0%) |
|  | Unknown | 34 (1.8%) | 39 (3.7%) | 25 (3.2%) | 27 (10.7%) | 95 (2.8%) |
| Geographic region, n (%) | Northeast | 321 (16.7%) | 179 (16.8%) | 123 (15.7%) | 48 (19.0%) | 597 (17.9%) |
|  | Midwest | 482 (25.1%) | 317 (29.7%) | 185 (23.7%) | 75 (29.8%) | 885 (26.5%) |
|  | South | 810 (42.2%) | 434 (40.7%) | 355 (45.5%) | 70 (27.8%) | 1,369 (40.9%) |
|  | West | 303 (15.8%) | 132 (12.4%) | 116 (14.9%) | 56 (22.2%) | 483 (14.4%) |
|  | Unknown | 3 (0.2%) | 4 (0.4%) | 2 (0.3%) | 3 (1.2%) | 10 (0.3%) |
| Episode length in days | Mean (SD) | 24.8 (19.6) | 37.1 (21.6) | 36.2 (21.9) | 35.2 (21.6) | 34.8 (22.2) |
|  | Median (Min, Max) | 15 (1, 115) | 34 (1, 60) | 32 (1, 60) | 25 (1, 60) | 27 (1, 115) |

Abbreviations: SD=standard deviation

Table 3.3. Baseline demographic characteristics, by comorbidity score

| Demographic characteristics | | NCI CCI score category: 0 (N=2,803) | NCI CCI score category: >0 - 2 (N=1,639) | NCI CCI score category: >2 (N=2,591) |
| --- | --- | --- | --- | --- |
| Age, years | Mean (SD) | 50.8 (16.0) | 56.1 (14.9) | 59.8 (12.1) |
|  | Median | 55 | 59 | 61 |
| Age category, years, n (%) | <18 | 150 (5.4%) | 61 (3.7%) | 30 (1.2%) |
|  | 18 to 59 | 1,727 (61.6%) | 782 (47.7%) | 1,071 (41.3%) |
|  | 60 to 74 | 850 (30.3%) | 677 (41.3%) | 1,251 (48.3%) |
|  | 75+ | 76 (2.7%) | 119 (7.3%) | 239 (9.2%) |
| Sex, n (%) | Male | 946 (33.7%) | 666 (40.6%) | 1,161 (44.8%) |
|  | Female | 1,857 (66.3%) | 973 (59.4%) | 1,430 (55.2%) |
| Payer type, n (%) | Commercial | 2,579 (92.0%) | 1,430 (87.2%) | 2,148 (82.9%) |
|  | Medicare | 98 (3.5%) | 121 (7.4%) | 299 (11.5%) |
|  | Medicaid | 59 (2.1%) | 29 (1.8%) | 62 (2.4%) |
|  | Unknown | 67 (2.4%) | 59 (3.6%) | 82 (3.2%) |
| Geographic region, n (%) | Northeast | 529 (18.9%) | 272 (16.6%) | 447 (17.3%) |
|  | Midwest | 729 (26.0%) | 447 (27.3%) | 708 (27.3%) |
|  | South | 1,118 (39.9%) | 635 (38.7%) | 1,062 (41.0%) |
|  | West | 416 (14.8%) | 283 (17.3%) | 365 (14.1%) |
|  | Unknown | 11 (0.4%) | 2 (0.1%) | 9 (0.3%) |
| Episode length in days | Mean (SD) | 29.2 (21.5) | 31.7 (21.5) | 34.5 (21.9) |
|  | Median (Min, Max) | 18 (1, 115) | 21 (1, 60) | 26 (1, 60) |

*Abbreviations: CCI=Charlson comorbidity index; NCI=National Cancer Institute; SD=standard deviation*

Table 3.4. Baseline demographic characteristics, by age category

| Demographic characteristics | | Age category: <18  (N=241) | Age category: 18 to 59 (N=3,580) | Age category: 60 to 74 (N=2,778) | Age category: 75+  (N=434) |
| --- | --- | --- | --- | --- | --- |
| Age, years | Mean (SD) | 8.9 (5.2) | 48.5 (9.8) | 64.5 (3.7) | 79.8 (3.8) |
|  | Median | 9 | 52 | 64 | 79 |
| Age category, years, n (%) | <18 | 241 (100.0%) | 0 (0.0%) | 0 (0.0%) | 0 (0.0%) |
|  | 18 to 59 | 0 (0.0%) | 3,580 (100.0%) | 0 (0.0%) | 0 (0.0%) |
|  | 60 to 74 | 0 (0.0%) | 0 (0.0%) | 2,778 (100.0%) | 0 (0.0%) |
|  | 75+ | 0 (0.0%) | 0 (0.0%) | 0 (0.0%) | 434 (100.0%) |
| Sex, n (%) | Male | 131 (54.4%) | 1,233 (34.4%) | 1,163 (41.9%) | 246 (56.7%) |
|  | Female | 110 (45.6%) | 2,347 (65.6%) | 1,615 (58.1%) | 188 (43.3%) |
| Payer type, n (%) | Commercial | 230 (95.4%) | 3,447 (96.3%) | 2,358 (84.9%) | 122 (28.1%) |
|  | Medicare | 0 (0.0%) | 22 (0.6%) | 296 (10.7%) | 200 (46.1%) |
|  | Medicaid | 11 (4.6%) | 96 (2.7%) | 40 (1.4%) | 3 (0.7%) |
|  | Unknown | 0 (0.0%) | 15 (0.4%) | 84 (3.0%) | 109 (25.1%) |
| Geographic region, n (%) | Northeast | 45 (18.7%) | 599 (16.7%) | 518 (18.6%) | 86 (19.8%) |
|  | Midwest | 77 (32.0%) | 930 (26.0%) | 727 (26.2%) | 150 (34.6%) |
|  | South | 90 (37.3%) | 1,543 (43.1%) | 1,096 (39.5%) | 86 (19.8%) |
|  | West | 29 (12.0%) | 496 (13.9%) | 427 (15.4%) | 112 (25.8%) |
|  | Unknown | 0 (0.0%) | 12 (0.3%) | 10 (0.4%) | 0 (0.0%) |
| Episode length in days | Mean (SD) | 28.2 (19.8) | 28.8 (21.3) | 34.4 (21.9) | 40.7 (21.3) |
|  | Median (Min, Max) | 22 (1, 60) | 18 (1, 115) | 26 (1, 60) | 60 (1, 60) |

*Abbreviations: SD=standard deviation*
